# Meta-analysis of urbanization impact on rainfall modification

**DOI:** 10.1038/s41598-019-42494-2

**Published:** 2019-05-13

**Authors:** Jie Liu, Dev Niyogi

**Affiliations:** 1 Department of Earth, Atmospheric, and Planetary Sciences, Purdue University, West Lafayette, IN 47907 USA; 2 Departments of Statistics, Purdue University, West Lafayette, IN 47907 USA; 3 Department of Agronomy – Crops, Soils, and Water Sciences, Purdue University, West Lafayette, IN 47907 USA

**Keywords:** Hydrology, Environmental impact

## Abstract

Even though it is known that urbanization affects rainfall, studies vary regarding the magnitude and location of rainfall change. To develop a comprehensive understanding of rainfall modification due to urbanization, a systematic meta-analysis is undertaken. The initial search identified over 2000 papers of which 489 were carefully analyzed. From these papers, 85 studies from 48 papers could be used in a quantitative meta-analysis assessment. Results were analyzed for case studies versus climatological assessments, observational versus modeling studies and for day versus night. Results highlight that urbanization modifies rainfall, such that mean precipitation is enhanced by 18% downwind of the city, 16% over the city, 2% on the left and 4% on the right with respect to the storm direction. The rainfall enhancement occurred approximately 20–50 km from the city center. Study results help develop a more complete picture of the role of urban processes in rainfall modification and highlight that rainfall increases not only downwind of the city but also over the city. These findings have implications for urban flooding as well as hydroclimatological studies. This meta-analysis highlights the need for standardizing how the results are presented in future studies to aid the generalization of findings.

## Introduction

Recent decades have witnessed a dramatic increase in urbanization and resulting land use/cover change. It is recognized that while only about 1% of the land can be regarded as urban area, the impacts affect a large population and are socioeconomically important. Further urbanization impacts are expected to increase in terms of the spatial coverage and density and at a faster rate in the future^1^. Urbanization not only changes the surface energy balance through modified surface albedo and heat storage, but it also contributes to regional pollution, anthropogenic emissions, and heat release.

Cities can have a notable impact on the local and regional climate. A well-known feature of such change is the so-called ‘urban heat island’ (UHI), where urban areas are warmer than the surrounding rural areas typically by about 1–3 degree Celsius^2–4^. This understanding of urban impacts on temperature has matured and is also used in the development of urban climate mitigation strategies including green buildings and the design of green spaces^5^.

While the temperature effects due to urbanization are well studied and understood, the effect of landscape feedback on rainfall is evolving. This is because the rainfall changes are dynamic and depend on a number of other factors^6,7^. For the urban landscape, the impact is not collocated and depends on the wind; and heating at the surface and the boundary layer due to surface characteristics, as well as aerosols above the urban surface. The impact also depends on urban – rural surface flux gradients and the moisture availability in the rural area. Early work by Horton^8^ discussed the potential for rainfall modification due to New York City. A more definitive evidence was reported after several decades from the Metropolitan Meteorological Experiment (METROMEX)^9^ conducted in St. Louis, MO, USA, in the 1970s. The METROMEX results found rainfall increase by 10–17% downwind of the city. Reviews by Landsberg^7^ and Shepherd^1^, and a series of studies following them^10–14^ have provided increased confidence in the findings that urbanization has a notable impact on rainfall changes.

Despite the growing knowledge about urban rainfall modification, a quantitative assessment and analysis is lacking. This is because, the same urban area can yield different rainfall effects due to dynamical environmental factors related to aerosol emissions, surface and boundary layer feedbacks, mesoscale convergence, and thermodynamic considerations. An objective assessment is however, increasingly important as cities continue being vulnerable to rainfall extremes witnessing both floods^15^ and droughts^16–18^, and an emerging topic of interest is how precipitation changes are affected by the urban environment^19,20^.

A study by Niyogi *et al*.^21^ reviewed 96 summer storms spanning over a decade for the Indianapolis urban area, using radar and multiscale rainfall datasets. Their results showed that the majority of thunderstorms (approximately 60%) over the urban area showed structural and morphological changes as compared to approximately 25% of the storms that showed similar changes over the surrounding rural landscape. The study also reported different impacts of urbanization on storm behavior for daytime and nighttime events. Of the observed storms, an overwhelming 71% of the daytime storms showed urbanization impact (as compared to 42% for night). Interestingly, the study found that the storms tend to bifurcate or split when they approach the urban area and reemerge downwind of the city as a more powerful storm.

A number of studies show rainfall not only increases downwind of cities but can also increase along the lateral edges, and sometimes even over the city center. Examples include studies on rainfall modification around Beijing city (e.g.^22,23^). The Yang *et al*. study^23^, documented increased rainfall downwind of Beijing while for the same region, Dou *et al*.^22^ reported reduced rainfall over and downwind of the city. This reduction was as much as 35% downwind, compensated by an increase in rainfall (by about 15%) along the lateral edges, and was likely due to different UHI intensity between the Yang *et al*. and the Dou *et al*. study.

Another variability in urban-rainfall modification is due to aerosols. In a modeling study involving the effect of urban aerosols on downwind rainfall modification, van den Heever and Cotton^24^ contended that the mechanisms associated with urban rainfall modification are still not well understood and their relative importance is not clear. The urban enhancement of rainfall can depend on aerosol loading under low background aerosol concentrations. For areas with low background aerosol concentrations, addition of urban aerosols can have a notable impact on convective storms and associated rainfall. More generally, studies highlight nonlinear relationship between aerosols, cloud microphysics, and surface roughness as well as UHI-driven storm dynamics which can affect urban convection and precipitation.

Thus, despite a growing number of published studies that documented precipitation modification by urbanization, their conclusions differ in terms of how and how much does the rainfall change downwind or upwind of the city. Most studies highlight some precipitation intensification downwind of urban areas due to urbanization^25–29^. Some published studies concluded that urban areas dissipated the thunderstorm passing over the cities, and there was more precipitation upwind of the city^21,30^. On the other hand, some studies^21,22^ found that the areas adjacent to the cities get more rainfall than other locations.

It is important to recognize that variability in the reported results is also likely due to the different challenges associated with quantification of urbanization impacts in both observational and modeling studies. In particular, there is no standardized mode of conducting the experimental design and setup of control versus the experimental dataset. In some papers, a comparison between the precipitation difference (change) with its surroundings is undertaken while some studies take the rainfall anomaly for a different climatological normal (e.g., about 30 years prior) as the control group. Typically, to analyze urbanization impacts on precipitation, the control group is the landscape without urban area in the same location. However, it is not possible for observational studies to set twin experiments with identical settings^31^. Therefore, a neighboring non-urban location is often taken as the control e.g.^9^. In the modeling study, the urban region is typically replaced by the nonurban landscape and the impacts are assessed e.g.^21,26^.

Another example of the uncertainty is the modeling framework and the processes being considered (e.g., some consider aerosols explicitly^24^, while several modeling studies ignore them or have implicit consideration)^21^. There are also grid spacing (model resolution), cloud convection, and microphysics parameterization-related dependency on the rainfall simulation in the model and the results have an implicit bias in simulating the true precipitation change. It is also noteworthy that in most urban rainfall modification studies, no uncertainty analysis is reported (although some have resorted to ensemble assessment)^32^. Indeed, most papers provide a value of precipitation change based on one or two case studies with a single (non-ensemble) run of the best-performing model configuration. Thus, both observational and modeling studies reporting urban rainfall modification have inherent and various uncertainties. As a result, the findings related to urban rainfall modifications often need to be extracted by developing a clear dynamical understanding of the feedbacks^33^. Using only a statistical analysis can potentially yield incorrect conclusions (as discussed in^34^).

This is another reason that a standardized meta-analysis pursued here is necessary to review the results in a common framework.

Recognizing such variability in urban studies, a U.S. National Research Council report^25^ highlighted that: “the literature has clearly established that precipitation may be affected by urban regions. Most operational organizations (and climate resiliency groups) are likely not aware of this conclusion or may be skeptical. The issue is further complicated by the uncertainty in the sign of the effect and under what conditions the effects are most evident”.

Thus, urban rainfall modification can be due to urban heat island interactions, urban roughness effects, and aerosols, each of which can have a positive, negative, or synergistic impact^25^. In the wake of these diverse findings and conclusions, and because of the availability of an increasing number of peer-reviewed publications (see Supplementary Fig. 1) as well as the maturity of the results, there are several science questions relevant to the issue of urban rainfall modification. Some of these are stated as:

1. Are urban areas modifying rainfall? If yes, by what amount and where is the modification occurring?
2. What is the mechanism or process (aerosols, urban heating, boundary layer dynamics due to surface heat flux gradients) causing urban rainfall change? What is the effect of aerosol types and concentrations?
3. Are there differences between the findings from case studies and climatology-based results? What is the impact of using different models and physics options on the urban rainfall results?
4. What is the effect of terrain on urban rainfall feedbacks? What is the effect of city size and shape?

As outlined in the Methods section, the current literature does not provide a robust sample of papers and quantitative results corresponding to different subclasses (e.g., aerosol forcing on urban rainfall climatology or urban rainfall modification in mountain terrain), to address each of these questions. As a result, only a subset of the questions for which we found better consistency in the sample were used to code into the dataset and develop our analysis.

Here, we seek to combine the results of different urban rainfall change studies and attempt a more definitive conclusion regarding precipitation impacts due to cities. This is undertaken by developing an objective meta-analysis of published studies. The selected papers quantified the urbanization impact on precipitation. Lowry^35^ has presented one example of a problem analysis framework for studying the urban rainfall issue. In his study, the estimates of rainfall changes are categorized for differences between urban-rural magnitude change, upwind downwind rainfall change, changes in ratio of urban versus regional rainfall, temporal changes in the trends of rainfall differences or ratios, and weekday versus weekend rainfall changes. For most of the studies reviewed, the issue could be analyzed in the context of the urban – rural magnitude changes and location; i.e., upwind, downwind, and rainfall change. Study details are outlined in the following sections.

## Results

### Summary of different papers and studies

**Figure 1:**
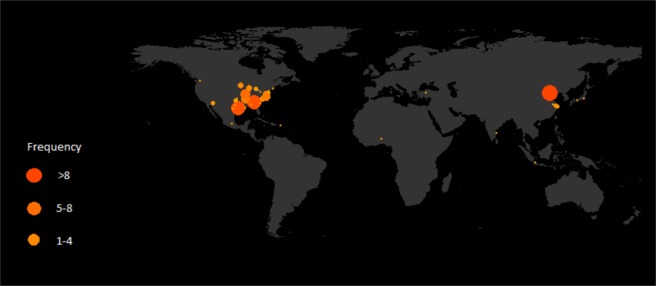
Location map of analyzed cities.

Table 1 shows the classification of the coded articles included in this analysis. It is important to note that despite over 489 papers on this topic that were shortlisted from over 2000 initially identified, only a small fraction (48) meet the quantitative criteria set by meta-analysis. The 48 papers covered 85 studies or assessments. For example, a paper which had analysis about climatology of urban rainfall and also discussed select case studies is counted as one paper but two studies. The majority of papers provide a statistical or summary perspective regarding how much and where the rainfall has been changing. Fortunately, the papers that fit the meta-analysis criteria are highly representative of the larger population of those published. For example, we found that both the larger population and the meta-analysis subset are mostly for summer, and most studies do not separate daytime and nighttime storms when conducting the analysis. This issue is also discussed in the previous section and is one of the reasons we were motivated to develop the meta-analysis. From Fig. 1, we see that the cities for which analysis exists are located primarily in the USA and China. In particular, Beijing, China has been analyzed eight times (16% of the sample) while Atlanta, USA has been analyzed in seven papers (14% of the sample). Most studies are over highly developed inland cities, but some coastal cities (or near the Great Lakes) such as Shanghai, Chicago, Mumbai and Tokyo also have been studied. We also found that there are some studies about the impact of the urban cluster on precipitation from the Hangzhou-Changzhou-Suzhou cluster in China, or the New York-New Jersey, and Baltimore - Washington D.C. clusters as well as the inner-land city clusters of Raleigh-Cary or the Minneapolis – St. Paul area. However, cities with mountainous terrain are lacking detailed analysis, and only Taipei is analyzed in the mountain city group (and more recently analysis over San Miguel de Tucuman in northwest Argentina^12^ has become available). Some studies have been conducted over large domains spanning many cities (e.g., Eastern US^36^). Figure 1 shows the location of the analyzed cities. The cities being studied are mostly located in North America and China; Beijing and Atlanta have been studied seven and eight times, respectively.

**Table 1:** Classification of coded articles.

Climatological studies	Observational studies	Winter	Day	
			Night	
			All day	(Han, 2014)
		Summer	Day	(Ochoa, 2015) (Ganeshan, 2013)
			Night	(Burian, 2005)
			All day	(Zhang, 2014) (Yeung, 2011) (Hu, 2015) (Zhang, 2009) (Keuser, 2014) (Diem, 2005) (Huff, 1973) (Changnon, 1979) (Wright, 2012) (Shepherd, 2002) (Yang, 2014) (Hand, 2009) (Efe, 2013) (Cicek, 2005) (Yeung, 2015) (Shepherd, 2006) (Hayes, 2008) (Daniels, 2015) (Huff, 1975) (Chow, 1984)
		Others		(Sanderson, 1973) (Paulikas, 2014) (Trevino, 2012)
	Modeling Studies	Winter	Day	
			Night	
			All day	
		Summer	Day	(Ochoa, 2015)
			Night	(Wichansky, 2008) (Dou, 2015)
			All day	(Yang and Tian, 2014) (Yang, 2012) (Shimadera, 2015) (Argueso, 2016) (Zhang, 2014) (Yeung, 2011) (Hu, 2015)
Case studies	Observational studies	Winter	Day	
			Night	
			Others	
		Summer	Day	(Ntelekos, 2008) (Simpson, 2009)
			Night	(Mote, 2007)
			Others	
		Others	NA	
	Modeling studies	Winter	Day	
			Night	
			Others	(Comarazamy, 2010) (Perryman, 2013)
		Summer	Day	(Shem, 2009) (Ntelekos, 2008)
			Night	(Li, 2013) (Grossman, 2011) (Yang, 2014) (Souma, 2013) (Heever, 2007) (carri, 2010)
			Others	(Ma, 2015) (Zhong, 2015) (Bornstein, 2012)

Thus, it is noted that there is limited geographical diversity in the study regions, and most of the studies are conducted over relatively benign terrain (with the exception of a few papers in coastal regions or for cities with complex topography). The analysis potentially has this implicit bias in the results. We do not believe there is a city-specific bias in the conclusions that have emerged. In other words, the processes and findings over Beijing for example, are comparable to those found over Indianapolis, Atlanta, or New York City. As a result, just like boundary layer features have been found to be geographically invariant, we believe that the urban feedbacks, which are closely tied to boundary layer processes and mesoscale convergence/divergence patterns (as well as aerosols interactions), are geographically insensitive and the findings and conclusions are transferable.

Here we first summarize the different grouping for the reported studies. The studies can be classified as under two groups- those based off climatological assessments or those involving case studies. Then for each of these groups, three additional subgroups were done. The first was with respect to studies based off modeling analysis or those from observations alone. The second subgroup considered studies reporting results for daytime versus nocturnal storms. A third grouping was attempted considering summer versus winter conditions. Table 2 summarizes the number of studies for each of these grouping.

**Table 2:** Frequency table for case and climatology based studies.

Case Studies						Climatological Studies					
Method	Number of studies	Diurnal	Number of studies	Season	Number of studies	Method	Number of studies	Diurnal	Number of studies	Season	Number of studies
model	14	day	6	summer	16	model	11	day	8	summer	51
observation	4	night	6	winter	2	observation	54	night	5	winter	8
		combined analysis	6					combined analysis	52		

The subgroups-based analysis was only done for the climatological studies in consideration of the sample size.

It is noted that the majority of studies involve climatological assessment of urban storms and rainfall (18 case studies versus 65 climatology based). We find that, for the group involving the case studies, almost all the events corresponded to summer storms (and only two studies are for winter); therefore, even though the winter-time results are deemed scientifically valid, the seasonal grouping cannot be considered statistically reliable, and hence not discussed further. Similarly, the model versus observational subgroup for the case studies had a limited sample size (only 4 studies for observational studies) and no additional analysis was performed for that subgroup. Furthermore, the day versus night subgrouping involving the case studies had 6 studies for day and night respectively, and is considered as reasonable in the context of the representative nature of these studies. However, on further separating the studies with regrading to location, there is less than 3 studies for each location (up, down, left, right and center of the city), and this is considered unreliable for conducting additional statistics and not analyzed further. To summarize, no additional subgrouping based analysis (case versus climatology, day versus night and summer versus winter) for case study based subgroup was done because of small sample size.

We highlight that these subgroups are scientifically important to study and further research is needed to develop this body of knowledge. To summarize this further, (i) for the quantitative statistical part, we group the case and climatology together and calculate the summary effects (by summarizing the precipitation change in different locations such as upwind, downwind of the city) based on all the studies. This result is summarized in the following subsection: “Overall Result”. (ii) However, considering the perspective as regards to the manner in which these studies are developed and the analysis undertaken in the context of meteorological considerations, we recognize climatology and case studies as separate group of studies. Therefore, additional grouping is done for case studies versus climatology. We summarized the precipitation change for both subgroups: case and climatology. The results has been presented in subsection: Case studies versus Climatology; (iii) Further, we separated the analysis regarding case studies and climatological precipitation under each subgroups of summer versus winter, observation versus model, day versus night. Indeed, because of the sample size considerations, we restrict the quantitative meta-analysis for climatological studies only, and resort to descriptive discussion with respect to the case studies. That is, the day versus night or model versus observation sub grouping was done only for climatological studies and not for the papers related to case studies. These results are presented as in subsection: Model versus Observational studies and Day versus Night.

### Overall Results

An ANOVA was performed for the different studies considering following main-effects: (a) Method: model and observations; (b) Event: case and climatology; (c) Diurnal: day versus night; (d) Season: Summer and Winter; and (e) Location of the rainfall change: Upwind, Downwind, Center, Right side or Left side of the city. All the 85 studies from 48 papers are included in the ANOVA. Two sided F-test is used with significance level of 0.05. Results indicated that only location of precipitation change as a main-effect is significant (p value 0.0162). That is results are not different based on any other discrimination or grouping except for the change in rainfall with respect to location: Upwind, Downwind, Center, Right and Left. This is important to help cluster the studies and to focus on the significant main-effect. As a result, we combined the 85 studies from 48 coded articles to assess the rainfall change. We highlight again that, while from the statistical perspective these studies emerge as similar, from the meteorological consideration, there is value in reviewing the results from the different subgrouping. So these results will be presented from the perspective of a review to summarize what is found from the different studies and not considered significant in the context of a statistical meta-analysis.

**Figure 2:**
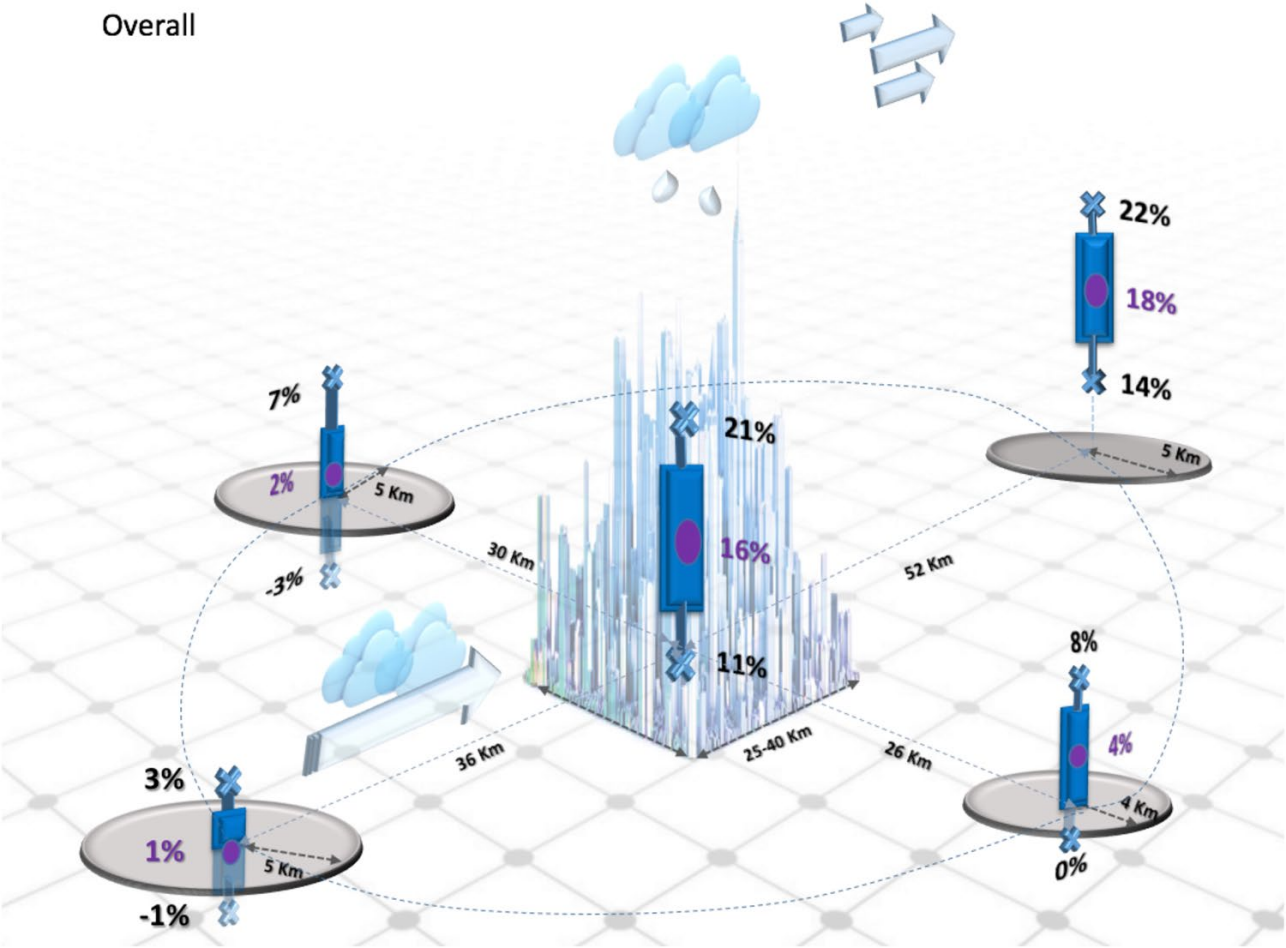
Precipitation changes over urban areas and for surrounding landscape. The bars indicate the sample standard deviation for the precipitation change, and circles correspond to the mean change in precipitation location. On average, urban areas and the surrounding region experienced precipitation increases. The largest signal noted in a number of studies, was prominently in the downwind region of the city and experienced the highest rainfall change: 18% increase on average, (a range of 14 to 22% with one standard deviation). The distance over which these changes occurred (mostly increases in rainfall) is approximately 52 km downwind, and about 31 to 41 km upwind.

Figure 2 and Table 3 show that the city and its surrounding region can experience precipitation modification (typically an increase). The largest signal, as noted in a number of studies, is prominently downwind of the city and experiences the highest rainfall change: 18% increase on average, (with a sample standard deviation of 4%). While the downwind intensification of rainfall is well characterized in the literature, the meta-analysis results also show a robust signature of an upwind increase in rainfall in the different studies. The distance over which these changes occur (mainly increased rainfall) is approximately 52 km downwind, and about 31 to 41 km upwind. There is also a notable signature in some studies (e.g.^22^) regarding a rainfall increase laterally (peripheral) to the city. Results indicate that the increase is noted approximately 30 km on the left and 26 km on the right (with respect to the storm direction). Whether these two distances are statistically different or have any particular dynamic significance (that is, whether the right side of the storm is more impacted due to the anticyclonic low-pressure system over the city and hence the distance is farther away as compared to the left side of the city), is not apparent.

**Table 3:** Meta-analysis summary results.

	Rainfall change percentage			Rainfall change location (km)		
	Mean Effect Size (*ES*_*V*_)	Mean Effect size ± Standard Deviation $({\boldsymbol{E}}{{\boldsymbol{S}}}_{{\boldsymbol{V}}}\pm {\boldsymbol{S}}{{\boldsymbol{E}}}_{{\boldsymbol{E}}{{\boldsymbol{S}}}_{{\boldsymbol{V}}}})$	Number of studies	Mean Effect Size (*ES*_*V*_)	Mean Effect size ± Standard Deviation $({\boldsymbol{E}}{{\boldsymbol{S}}}_{{\boldsymbol{V}}}\pm {\boldsymbol{S}}{{\boldsymbol{E}}}_{{\boldsymbol{E}}{{\boldsymbol{S}}}_{{\boldsymbol{V}}}})$	Number of studies
Urban Center	16	[11, 21]	54	NA	NA	NA
Upwind	1	[−1, 3]	28	36	[31, 41]	11
Downwind	18	[14, 22]	64	52	[47, 57]	47
Left Side	2	[−3, 7]	24	30	[25, 35]	11
Right Side	4	[0, 8]	23	26	[22, 30]	13

### Case studies versus Climatology

Tables 4 and 5 show the meta-analysis results for precipitation changes due to urban feedback as revealed from case study versus climatology-based studies. It is interesting to note that the results from the case studies may not always be indicative of the climatological findings. For example, reviewing the case studies, the results indicate urban precipitation increases downwind and urban center by about 19% and 3% respectively, along with some lateral (left) increase (by about 10%). The climatological results, while also indicating a downwind increase (by about 18%), show a significant increase over the city (about 20%) as well as an increase in rainfall on the right and left (by about 9% and 7%). In fact, the upwind side of the city shows a reduction in rainfall in the case studies but an increase in the climatology studies. This lack of agreement for rainfall changes observed in case studies versus climatological analysis is perplexing but may be explained in the manner in which case studies are often performed. For example, it is likely that the cases selected are often extreme or otherwise noteworthy event, where a clear signature in the process is expected, or for which special observations are available. Also, case studies may often be selected to “prove” or explain the urban downwind modification feature and thus implicitly bias the analysis and the results. This anomaly between the case study versus climatology needs to be examined further because case study findings are often assumed as representative of the broader context and this may not be the case.

**Table 4:** Meta-analysis subgroup results: Case studies.

	Rainfall change percentage			Rainfall change location (km)		
	Mean Effect Size (*ES*_*V*_)	Mean Effect size ± Standard Deviation $({\boldsymbol{E}}{{\boldsymbol{S}}}_{{\boldsymbol{V}}}\pm {\boldsymbol{S}}{{\boldsymbol{E}}}_{{\boldsymbol{E}}{{\boldsymbol{S}}}_{{\boldsymbol{V}}}})$	Number of studies	Mean Effect Size (*ES*_*V*_)	Mean Effect size ± Standard Deviation $({\boldsymbol{E}}{{\boldsymbol{S}}}_{{\boldsymbol{V}}}\pm {\boldsymbol{S}}{{\boldsymbol{E}}}_{{\boldsymbol{E}}{{\boldsymbol{S}}}_{{\boldsymbol{V}}}})$	Number of studies
Urban Center	3	[−9, 15]	10	NA	NA	NA
Upwind	−7	[−13, −1]	9	30	[26, 34]	4
Downwind	19	[12, 26]	16	63	[50, 76]	15
Left Side	10	[2, 18]	9	20	[14, 26]	3
Right Side	2	[−4, 8]	8	35	[30, 40]	2

**Table 5:** Meta-analysis subgroup results: Climatology studies.

	Rainfall change percentage			Rainfall change location (km)		
	Mean Effect Size (*ES*_*V*_)	Mean Effect size ± Standard Deviation $({\boldsymbol{E}}{{\boldsymbol{S}}}_{{\boldsymbol{V}}}\pm {\boldsymbol{S}}{{\boldsymbol{E}}}_{{\boldsymbol{E}}{{\boldsymbol{S}}}_{{\boldsymbol{V}}}})$	Number of studies	Mean Effect Size (*ES*_*V*_)	Mean Effect size ± Standard Deviation $({\boldsymbol{E}}{{\boldsymbol{S}}}_{{\boldsymbol{V}}}\pm {\boldsymbol{S}}{{\boldsymbol{E}}}_{{\boldsymbol{E}}{{\boldsymbol{S}}}_{{\boldsymbol{V}}}})$	Number of studies
Urban Center	20	[15, 25]	44	NA	NA	NA
Upwind	2	[0, 4]	18	40	[32, 48]	7
Downwind	18	[14, 22]	48	47	[42, 52]	32
Left Side	7	[1, 13]	17	34	[27, 41]	8
Right Side	9	[4, 14]	16	24	[19, 29]	11

In both types of studies, rainfall increases are significant downwind as well as over the urban center.

### Model versus Observational studies

A statistically balanced dataset (in terms of the number of studies) was available for comparing the results of urbanization impacts from observational analysis versus model studies, from the climatological group. Tables 6 and 7 show the meta-analysis results comparing the findings.

**Table 6:** Meta-analysis subgroup results: Modeling (climatological studies).

	Rainfall change percentage			Rainfall change location (km)		
	Mean Effect Size (*ES*_*V*_)	Mean Effect size ± Standard Deviation $({\boldsymbol{E}}{{\boldsymbol{S}}}_{{\boldsymbol{V}}}\pm {\boldsymbol{S}}{{\boldsymbol{E}}}_{{\boldsymbol{E}}{{\boldsymbol{S}}}_{{\boldsymbol{V}}}})$	Number of studies	Mean Effect Size (*ES*_*V*_)	Mean Effect size ± Standard Deviation $({\boldsymbol{E}}{{\boldsymbol{S}}}_{{\boldsymbol{V}}}\pm {\boldsymbol{S}}{{\boldsymbol{E}}}_{{\boldsymbol{E}}{{\boldsymbol{S}}}_{{\boldsymbol{V}}}})$	Number of studies
Urban Center	20	[14, 26]	10	NA	NA	NA
Upwind	5	[3, 7]	7	28	[17, 39]	3
Downwind	0	[−7, 7]	8	25	[17, 33]	4
Left Side	1	[−2, 4]	7	15	[10, 20]	2
Right Side	7	[2, 12]	7	13	[8, 18]	4

**Table 7:** Meta-analysis subgroup results: Observational (climatological studies).

	Rainfall change percentage			Rainfall change location (km)		
	Mean Effect Size (*ES*_*V*_)	Mean Effect size ± Standard Deviation $({\boldsymbol{E}}{{\boldsymbol{S}}}_{{\boldsymbol{V}}}\pm {\boldsymbol{S}}{{\boldsymbol{E}}}_{{\boldsymbol{E}}{{\boldsymbol{S}}}_{{\boldsymbol{V}}}})$	Number of studies	Mean Effect Size (*ES*_*V*_)	Mean Effect size ± Standard Deviation $({\boldsymbol{E}}{{\boldsymbol{S}}}_{{\boldsymbol{V}}}\pm {\boldsymbol{S}}{{\boldsymbol{E}}}_{{\boldsymbol{E}}{{\boldsymbol{S}}}_{{\boldsymbol{V}}}})$	Number of studies
Urban Center	19	[13, 25]	34	NA	NA	NA
Upwind	0	[−2, 2]	12	47	[37, 57]	4
Downwind	22	[18, 26]	40	50	[45, 55]	28
Left Side	11	[1, 21]	11	41	[34, 48]	6
Right Side	10	[2, 18]	10	31	[26, 36]	7

It is interesting that observational studies show an increase in rainfall both at the center as well as downwind (19% and 22%), while model based climatological analysis tend to show a dominant increase over the center (by about 20%) and no significant increase downwind. The model results show a relatively smaller change due to urban feedback as compared to the observations. A recent multicity analysis of radar-derived precipitation changes over several U.S. cities^36^ shows that larger cities may have as much as a 25–50% increase in downwind thunderstorms (and hence rainfall potential). Results from meta-analysis also show that observational studies generally indicate a higher impact from urban areas as compared to the modeling studies. Whether this relatively muted response in the model is due to missing processes (e.g. aerosol and land - atmosphere feedback) or the way the results are analyzed (e.g., station data in observations versus grid-averaged results in models) or the resolution (grid spacing) in the models which does not discretize the city-center and the downwind effect for an urban grid is not clear^37^.

### Day versus Night

Tables 8 and 9 show the meta-analysis results for rainfall changes for storms occurring during the day versus night. Note that for the analysis, in consideration of the sample size, only the studies involving climatology are considered and the effects related to rainfall changes for downwind and over the city are discussed. In the analysis presented in Niyogi *et al*.^21^ for instance, it was shown that a larger fraction of daytime storms were impacted due to the urban heat island feedback. The heat island effect and the boundary layer dynamics appear to impact storm rainfall as an urban rural heterogeneity-based feedback. During the night, the land - atmospheric coupling is typically weaker as compared to the day, and as a result, the urban impact is also expected to be less dominant. The results from meta-analysis are consistent with this feature and show that for daytime cases, a significant increase in rainfall over the city (about 44% or more) and about a 16% increase downwind. For nighttime, the results show a different pattern with precipitation increase over the city by 14%, while downwind show significant precipitation deduction (to the order of 8%).

**Table 8:** Meta-analysis subgroup results: Daytime events.

	Rainfall change percentage			Rainfall change location (km)		
	Mean Effect Size (*ES*_*V*_)	Mean Effect size ± Standard Deviation $({\boldsymbol{E}}{{\boldsymbol{S}}}_{{\boldsymbol{V}}}\pm {\boldsymbol{S}}{{\boldsymbol{E}}}_{{\boldsymbol{E}}{{\boldsymbol{S}}}_{{\boldsymbol{V}}}})$	Number of studies	Mean Effect Size (*ES*_*V*_)	Mean Effect size ± Standard Deviation $({\boldsymbol{E}}{{\boldsymbol{S}}}_{{\boldsymbol{V}}}\pm {\boldsymbol{S}}{{\boldsymbol{E}}}_{{\boldsymbol{E}}{{\boldsymbol{S}}}_{{\boldsymbol{V}}}})$	Number of studies
Urban Center	44	[32, 56]	7	NA	NA	NA
Downwind	16	[5, 27]	7	30	[20, 40]	3

**Table 9:** Meta-analysis subgroup results: Nighttime events (climatological studies).

	Rainfall change percentage			Rainfall change location (km)		
	Mean Effect Size (*ES*_*V*_)	Mean Effect size ± Standard Deviation $({\boldsymbol{E}}{{\boldsymbol{S}}}_{{\boldsymbol{V}}}\pm {\boldsymbol{S}}{{\boldsymbol{E}}}_{{\boldsymbol{E}}{{\boldsymbol{S}}}_{{\boldsymbol{V}}}})$	Number of studies	Mean Effect Size (*ES*_*V*_)	Mean Effect size ± Standard Deviation $({\boldsymbol{E}}{{\boldsymbol{S}}}_{{\boldsymbol{V}}}\pm {\boldsymbol{S}}{{\boldsymbol{E}}}_{{\boldsymbol{E}}{{\boldsymbol{S}}}_{{\boldsymbol{V}}}})$	Number of studies
Urban Center	14	[−2, 30]	5	NA	NA	NA
Downwind	−8	[−25, 9]	4	NA	NA	NA

### Summary of the urban-rainfall processes

Combining the different studies, the understanding that emerges regarding the processes and mechanisms leading to changes in urban rainfall is summarized here. It is interesting to note that a summary provided in Huff and Changnon^13^ still largely holds true. In their work, they document four potential pathways for urban rainfall modification. These include (i) a thermal effect due to atmospheric instability over the urban area due to the heat island; (ii) a barrier effect caused by possible air flow obstruction around the urban area along with increased mechanical turbulence in the lower boundary layer; (iii) urban aerosol effects, that is modifications due to aerosols and pollution leading to changes in the heating profiles, clouds and microphysical process over urban areas, and (iv) modification of the lower boundary layer moisture and thermal characteristics due to anthropogenic sources such as cooling towers, and urban evaporation from greenspaces.

The different papers reviewed provide some common features associated with urban rainfall modification that fall along the above-listed four effects. For instance, Chow and Chang^38^ list three of the four of these effects (except for anthropogenic heating) as possible mechanisms for rainfall changes around Shanghai. Some studies^21,26,39^ find that the urban-rural land cover gradient enhances vertical mixing, while convergence over or around the city along with enhanced vertical velocities aid convection in and around urban areas. There is not only increased local convergence but also a prominent role of moisture advection over the city modifying the rainfall. This was noted in both radar observations as well as modeling studies^32,40^. There is a potential for elevated and increased cloud water and rainfall water mixing ratios over urban areas, which can also have a significant role in the urban rainfall signature^41^. This change in atmospheric instability and convergence over urban areas is also considered to be a critical feature in wintertime urban precipitation modification^42^.

Urban roughness often leads to a change (reduction) in surface winds upwind of the city and a possible acceleration downwind. This leads to modified divergence and convergence patterns along the city boundaries and are reflective of the microscale lateral pressure gradients along the city transect. Thus, the physiographical heterogeneity in terms of the surface features manifests in the dynamical characteristics especially in the local winds. Studies such as^23^ report that the level of free convection and height of the planetary boundary layer are significantly increased over urban regions and maximum convective available potential energy is correspondingly decreased. The increased sensible heat flux from the urban surface plays a dominant role in the modification of simulated rainfall from a climatological perspective. In fact, for a study over Osaka, Japan^43^, precipitation increases over the city were generally due to enhancement of the formation and development of convective clouds attributed to an increase in sensible heat flux during the afternoon and evening periods.

Dou *et al*.^22^ discusses why some studies show increased rainfall over a city, while others report increases around or downwind. They note that low wind and high UHI settings can induce convergence into the city leading to higher rainfall totals over urban areas. On the other hand, high wind, low UHI events can create a barrier effect leading to intensified rain downwind and around the city (through storm bifurcation). This can often reduce rainfall over the city but increase it downwind.

One notable point of difference between urban rainfall studies is related to the dynamic and thermal role of urban roughness or heat island feedbacks as well as the role of urban aerosols in terms of their impact on rainfall modification. The issue becomes complicated because aerosol effects can align with the roughness or UHI-induced convergence regions and hence the dynamical, thermal, and aerosol feedbacks can be interactive and synergistic. Therefore, it is not surprising to find some studies reporting that one type of effect is more important than the other. For example, a study regarding rainfall changes in the Milwaukee-Lake Michigan region finds that surface processes may be important drivers and found no change in cloud aerosol properties over the urban area^33^. In a related paper over the Baltimore, Maryland and Washington DC region^25,44^, UHI was not found to be important especially for synoptically strong storms while aerosols were found to be more influential. In the climatological analysis of multi-cities^45^, UHI effects on urban rainfall modification were reported to be important for inland cities while for coastal cities, aerosols effects became more important.

Studies considering urban aerosol effects find that urban convergence is roughness and dynamics related and it is more vital than aerosol effects per se in creating convection over and away from the city^24^. However, once convection is triggered, the role of aerosols may be quite crucial in modifying the location, amount, and even the timing of urban rainfall-induced changes. It is important to note that when considering the role of urban aerosols, the relative concentrations with respect to rural background aerosols and urban–rural aerosol gradients also becomes important. Therefore, just as intense UHI or large differences in surface roughness can also cause dynamical changes in the regional convergence patterns, urban–rural aerosol differences can cause microphysical effects on urban clouds and convection thus affecting rainfall. Urban aerosols, especially in regions of low background aerosol regions such as coastal cities, can lead to higher and upper levels of the convective cells downwind of the city that are invigorated by a greater latent heat release linked to higher amounts of liquid water transported to supercooled levels. This invigoration may not always translate into increased rainfall, and under high aerosol concentrations, riming growth of ice particles is possible leading to reduced precipitation. Similar effects in terms of reduced urban rainfall and timing delays have been noted for increased urban pollution in relatively clean background regions^46^.

In summary, the various studies summarized in the meta-analysis show that the effects associated with urban rainfall modification can interactively enhance or reduce the anomaly. Therefore, it is still a challenge to detect the urban rainfall signatures within the observational and modeling studies. As mentioned, getting a pure urban effect (with and without urban land cover) is perhaps possible in the models but not in observations. Similarly, impacts that are dominated by one type of active process (thermal, roughness, or aerosol) may perhaps be a geographical feature. Nonetheless, all of these studies and the associated findings provide a robust conclusion that *urban areas modify rainfall*. The mechanism by which cities modify rainfall is also broadly understood. Dissecting and explicitly extracting the relative roles of different processes and the conditions under which one will become more dominant than the other is also evolving and still a challenge. Studies that assess urban rainfall modification using observations or models appears to provide a strong foundation that can be continued going forward in what appears to be a broadly agreed upon but self-organized framework by the community.

## Discussion

The impact of urbanization on precipitation was analyzed using the meta-analysis of 85 studies from 48 selected, representative published papers. By virtue of the design of the meta-analysis, all the papers selected showed that there was detectable impact of urbanization on rainfall. Several important summary findings emerge.

(i) Rainfall enhancement is found downwind *as well as* over the city. Considering all the studies included in the meta-analysis, results indicated that urban areas cause precipitation modification and the impact is highest downwind leading to an approximately 18% increase. A second and important feature that has emerged is that urbanization also increased rainfall over urban areas (city center) by 16% and this feature has not been sufficiently highlighted in urban rainfall modification studies. Study results also indicate lateral sides of the city could also have a slightly increased tendency for rainfall enhancement (about 2% to the left and about 4% to the right flank of the city).
(ii) Modeling results tend to underestimate the mean impact of urban rainfall modification compared to observational studies. Further, while observations indicate a potential for increased rainfall over and downwind of the city, the climatological modeling studies suggest a preferential increase over the city while case studies show preference for increase in rainfall downwind of the city with a modest increase over the city center.
(iii) There are differences in the urban feedback for rainfall events during day versus night. Based on the analysis of the climatological studies, there is a general (both day and night) increase in the rainfall over the city. Additionally, there is a notable increase in the downwind rainfall during the day, but a decrease in the downwind rain at night.
(iv) Considering all the 85 studies, these rainfall changes (typically increases) are approximately 52 km downwind, about 36 km upwind, and about 25–30 km when considering the right and left flank of the storms.

Thus, study results indicate that urbanization has a detectable and notable impact on regional rainfall characteristics. These summary findings are considered in developing a conceptual model regarding how cities affect rainfall (Fig. 2).

The unique contribution of this work lies in the first ever meta-analysis focusing on urban rainfall climatology and processes. Prior work has either used, traditional modeling approaches^34,47–49^ or an observed climatological synthesis to extract the urban - rainfall modification assessments. These reviews had subjective synthesis (i.e. a non-statistical threshold) inherent in traditional review approaches. Having conducted this meta-analysis, it is important to highlight that the results obtained are not simply a restatement of what was already previously known from some of the recent reviews or comprehensive studies (e.g.^1,6,21,22^). For example, (i) study results highlights the need for presenting results in a standard format the lack of which is a potential stumbling block in advancing the knowledge in this field; (ii) from the general conceptual model that appears in the literature, scientific understanding and popular conception is that rainfall intensifies downwind of the urban area and a number of schematic figures can be found documenting this viewpoint. It was therefore remarkable to see a strong and significant signal related to intensification of rainfall over the urban area and not just downwind. This does not mean that it was an unknown feature, only that it was not being highlighted or given credence in terms of the impacts associated with urban rainfall modification. *Our study strongly suggests that the impact of urban rainfall modification is as significant to rainfall modification over the city as it is downwind of the city*. This is an important conclusion that is possible because of this meta-analysis. (iii) We find notable differences and similarities in the results reported using observational versus modeling studies, and the climatological versus case-study based assessments. These features pave the way for developing future studies that need to consider why the models and observations differ in their outcome (reviewing the processes, method of analysis, and in terms of cases being studied). It is quite likely that case studies are selectively chosen to advance a certain perspective and test a particular feature and those results may not be applicable in terms of climatological changes expected due to urbanization. (iv) We have also been able to, for the first time, provide information regarding the uncertainty around the ‘point’ estimates of rainfall modification when comparing the results from different studies that use rainfall data from model grids versus rainfall from surface datasets.

We are not able to eliminate biases caused by the inability to attribute the rainfall as perfectly urban based or non-urban^7,31^; however, we eliminated the bias in the manner in which results were reported in the studies by resorting to the effect-size calculation. In other review papers, typically the numerical results reported in the research were compared. In this paper, we studied the original tables and figures in those papers, and quantified the precipitation change from the original papers instead of only taking the quantitative results reported in the conclusions. A standard pipeline and design of experiments is needed when reporting results due to urban rainfall modification. However, considering the modeling uncertainty, we recommend that studies should conduct more simulations, and provide the mean change along with the uncertainty (i.e., standard deviation or confidence interval). In our paper, we extracted the original data from several papers conducting modeling studies, so were able to report the mean change along with the standard deviation.

Additional limitation of the work lies in the surprisingly small sample size that qualified for inclusion within the meta-analysis criteria. While almost 500 papers were initially identified, only about 10% had quantitative information that could be used in an objective manner to develop the meta-analytical assessment. This highlights the point mentioned previously regarding the *need for future studies to have a structure in place where rainfall modification* (*increase or decrease*) *for upwind, downwind, over the city, and left and right of the storm is explicitly stated. Also needed, is the distance with respect to the definition of these locations*. Adding this information will help urban rainfall climatology research to progress further in translating and transferring the findings from different studies to a common understanding. Such ‘standardization’ of the reporting of the urban impacts will also increasing confidence in the findings, and expand their use to engineering applications needed for urban planning and hazard mitigation such as from floods.

Despite the limitations, we believe that the number of papers that were available for the meta-analysis are qualitatively rich in terms of their content with many useful broad, common findings. As a result, we believe these papers yield sufficiently accurate and representative knowledge regarding the current state of the field of urban rainfall modification. Indeed, had the results from these different papers been more diverse, i.e., not having commonality in the findings or models or approaches, we suspect a much large sample size would have been necessary. Based on either our analysis or from the literature review, it is not possible to guesstimate what a good sample size might be. However, from the statistical design of the experiment, we believe that the sample was adequate to capture the combinations of- observational versus modeling studies, case studies versus climatology. We highlight that the available literature and sample size is likely not adequate or robust enough for urban aerosol-based climatology versus case studies, for studying the role of city size and shape on storm characteristics, or for determining the role of topography on urban rainfall modification. As a result, the discussion presented here has excluded these aspects even though they were part of the initial analysis framework.

Overall, the results suggest there is high fidelity to the notion that *urban areas affect rainfall characteristics in terms of their intensity and location*. These features have helped develop a quantitative and graphical representation which can be evolved in future studies.

## Methods

Meta-analysis is a powerful method used for quantitative literature review^50,51^. The classical reviews often tend to develop a qualitative subjective description of the process or the phenomenon^52–56^. Such reviews are valuable for summarization of the research (e.g.^1,53,56^) but are not designed to provide a quantitative synthesis of the effects being studied. Meta-analysis is employed as a class of review where potentially a more precise, objective estimation of results from available studies is possible. By using the meta-analytic approach to do a systematic review, the statistical power is greatly improved compared to simply drawing conclusions from every study. An obvious disadvantage of meta-analysis, as compared to the traditional review, is that it narrows the number of studies that can fit the quantitative, review criteria that are set for developing the study.

### Identification

Our meta-analysis method for urbanization and magnitudes of precipitation change quantitatively combines and summarizes research results across individual and independent papers published in peer-review journals and conference. The first step in a meta-analysis is to find all the pertinent articles on this topic. We used a key word search and expert recommendations to find the related papers. The 48 papers that were selected are indexed as^10,13,22–24,26–29,32,33,38–46,57–84^. The detailed identification methods are described in the Supplementary Materials.

### Abstraction

The initial search yielded more than 2000 papers (Fig. S1 shown in Supplementary Materials). These papers were reviewed and initially screened for relevancy to the study topic. This list resulted in 489 papers, which were reviewed further to identify papers that explicitly quantify the amount as well as location of anomalous precipitation. The quantitative review follows a structured protocol, which includes presetting objectives and the inclusion criteria for studies, approach for data collection, and the analyses to be done.

In the assessment undertaken, the following criteria needed to be met to be included for meta-analysis:

1. The study area in the paper must be at the city, metropolitan, or regional scale (city clusters) (less than 100,000 km^2^).
2. The study must quantify the accumulated rainfall anomaly in a specific location over a specific period of time (e.g., a 30% precipitation increase 50 km downwind of Atlanta annually for the climatology study, daily for case studies).
3. The study must be original research (and not a review of prior work).
4. The study must have been published in an English-language source.

After applying the criteria to the original 489 papers, 48 papers met the criteria and were selected for meta-analysis (information regarding these 48 papers is listed in Supplementary materials). From the 48 papers, 85 studies have been coded. As mentioned, we distinguish a paper and study in terms of the analysis reported in the paper. So, a paper count corresponds to an individual paper, while studies means the case or climatology studies has been done for a specific location in a paper. A single paper can have more than one study.

### Coding of articles

After paper selection by the inclusion criteria, each paper was coded. This is a specific term from the meta-analysis perspective, meaning we identified the study and reviewed the data they used to develop the results. We used the subset of results relevant to our study and incorporated it into the broader database. In our case, we used information regarding where the rainfall changed and by what amount. This information is compiled and included in the larger synthesis dataset. The coding was done using the following: (1) reference; (2) year published; (3) cases included in the paper; (4) city studied; (5) analysis method (i.e., model or observation); (6) model name (e.g., Regional Atmospheric Modeling System (RAMS) or Weather Research and Forecasting (WRF) model); (7) data source; (8) study type (i.e., case studies or climatological); (9) urban center effect size (ES) (precipitation percentage changed in urban center); (10) sample size (i.e., how many years of data or events were analyzed in the study); (11) event size (i.e., how many cities or storms were analyzed); (12) downwind effect size (e.g., the percentage of downwind rainfall change); (13) distance between the urban center and the anomalous precipitation; and (14) other effect size (i.e., percentage of precipitation change at the center, upwind, left, and right side of the urban area).

The effect size is a standardized term, used here in the comparison of results across different papers. In our study, the effect size is defined by precipitation change as outlined per the coding above, and is calculated as:

1 $${\rm{ES}}=\frac{{{\rm{P}}}_{{\rm{u}}}-{{\rm{P}}}_{{\rm{nu}}}}{{{\rm{P}}}_{{\rm{nu}}}}$$

where the effect size is denoted as ES (precipitation change), P_u_ is the precipitation amount (mm) at a specific location (i.e., urban center or downwind) when urban area is present or under urban influence, while P_nu_ is the precipitation amount (mm) at that location when urban area is not present (in model studies) or away from the urban influence (in observational studies). If the ES is positive, it means there was a precipitation increase. The reviewed papers were selected to report the same quantity, which in this case was precipitation amount and change. If the rainfall amount was in a different unit (e.g., inches), we converted the values into mm, and then calculated the effect size of urban center, downwind, upwind, left, and right of a city. The effect size was thus for two specific precipitation attributes: (i) change in the amount, and (ii) change in the position. The period (i.e. daily or seasonal or annual) was based on the paper and subgroup being studied (outlined in Table 1). For case studies, it was typically based on daily data while for climatological studies, the rainfall volumes averaged over years/decades were typically taken.

### Meta-analysis

A meta-analysis was conducted with all data from the selected papers. The effect size of urban center, downwind, upwind, left, and right side was summarized. Then a statistical estimation of precipitation changes in the urban and surrounding area is quantified. The location of precipitation change is also further summarized. We categorized articles into groups to analyze the difference among groups, for (1) night or day, (2) model or observation analysis, (3) climatology or case study, and (4) winter or summer. As an example, for night or day comparison, we selected all studies from coded articles which analyzed the impact of urbanization on precipitation during the daytime, and compared against those during nighttime. We then conducted the analysis separately for these two groups and compared the results. The meta-analysis consists of two steps: homogeneous analysis and applying a summary model to articles.

#### Homogeneity analysis

After coding the articles, a homogeneity analysis was undertaken using the so-called ‘Q test’ to decide if the articles are consistent or not^50^ By consistent, we seek to determine if the results are similar or not. For example, one study might show a rainfall increase by 10%, 20 km downwind of the urban area; and another study might show that rainfall increases by 12% and 25 km downwind of the urban area. These two results are consistent and is fairly apparent. However, when we have a larger sample, we need some quantitative measurement, which is obtained by examining Q-test results. If the articles are consistent, the fixed effect model was applied, otherwise the random effect summary model was applied.

The analysis was conducted as follows. Each study is a unit of the meta-analysis. A Q-test was applied to measure heterogeneity among studies, in which a Q-value was determined and compared with tabulated critical values associated with a degree of freedom and the confidence interval desired (95% CI was used in this study).

2 $$SE=\sqrt{\frac{1}{n}}$$

3 $$w=n$$

4 $${I}^{2}=\frac{Q-df}{Q}$$

5 $$df=n-1$$

6 $$Q={\rm{\Sigma }}(w\cdot E{S}^{2})-\frac{{\rm{\Sigma }}(w\cdot S{E}^{2})}{{\rm{\Sigma }}(w)}$$

where *n* is the number of the studies and *w* is research duration. Other than *Q*, we also calculated the *I*^2^, which represents the heterogeneity and is calculated as a percentage of total variability in a set of effect size due to true heterogeneity. Equation (4) shows the formulation of the *I*^2^ calculation where *ES* is the effect size.

#### Random effect summary model

After effect size calculating and homogeneity analysis, we applied the effect summary model. If the null hypothesis is rejected, the random effect model was used since we know that differences exist in the sample population. The constant *V* is used and introduced in the random effects model to represent the variability in the population of effects. It is calculated following Eq. (7), as:

7 $$V=\frac{Q-df}{{\rm{\Sigma }}(w)-\frac{{\rm{\Sigma }}{w}^{2}}{\Sigma w}}$$

8 $${W}_{V}=\frac{1}{S{E}^{2}+V}$$

9 $$E{S}_{V}=\frac{\Sigma ({W}_{V}\cdot ES)}{\Sigma {W}_{V}}$$

10 $$S{E}_{E{S}_{V}}=\sqrt{\frac{1}{{\rm{\Sigma }}{W}_{V}}}$$

The effect summary is calculated as Eq. (9) and the standard error as Eq. (10). Assumption of this model is that there is one true effect size that underlies all the studies in the analysis, and the only difference is due to sampling errors.

The analysis is conducted in R with package metafor.

## Supplementary information

Suplemmentary
