# Supplementary material for: Meta-analysis of urbanization impact on rainfall modification
Source: Sci Rep. 2019 May 13;9:7301. doi: 10.1038/s41598-019-42494-2 (PMC6514167; doi:10.1038/s41598-019-42494-2)
Supplement: Supplementary file 1 — Suplemmentary [file 41598_2019_42494_MOESM1_ESM.docx]

Meta-analysis of urbanization impact on rainfall modification

Jie Liu1,2 and Dev Niyogi1,3

1 Department of Earth, Atmospheric, and Planetary Sciences

2 Departments of Statistics

3 Department of Agronomy

Purdue University, West Lafayette, IN, 47907, USA

**Supplementary materials**

A. SUMMARY OF IDENTIFICATION METHODS

A.1 Search method

1). Full search strings for literature search Common Keywords to all regions Topic= (urban OR urbanization OR cities OR city OR Urban induced OR Urban effect) AND Topic=( rainfall OR rain OR precipitation OR rainfall pattern ) AND Topic=( change OR increase OR growth OR modif* OR decrease OR extent).

2). Find studies through citation. Find papers citing the review by Shepherd, J.M., (2005) [1], and/or METROMEX study by Changnon et al. (1971) [9].

3). Add studies recommended by experts (e.g. [6], [22], [56]).

Refined by: Document Type= (ARTICLE OR ABSTRACT OR LETTER OR EDITORIAL) AND Languages=( ENGLISH )

B. SUMMARY OF PAPERS

B.1 Papers included in the meta-analysis

The search initially yielded more than 2000 papers. These papers were reviewed, starting with their abstracts and scanning the figures, tables, and the text of the paper. This lead to a shortlist of 489 papers that were relevant to the study topic, and were reviewed further. Of these, 48 papers had appropriate quantitative information and data related to the ability to assess how much is the change in rainfall due to the city/ urban area and where, with respect to the city, is the change occurring.

C. URBAN RAINFALL RELATED PUBLISHED PAPERS


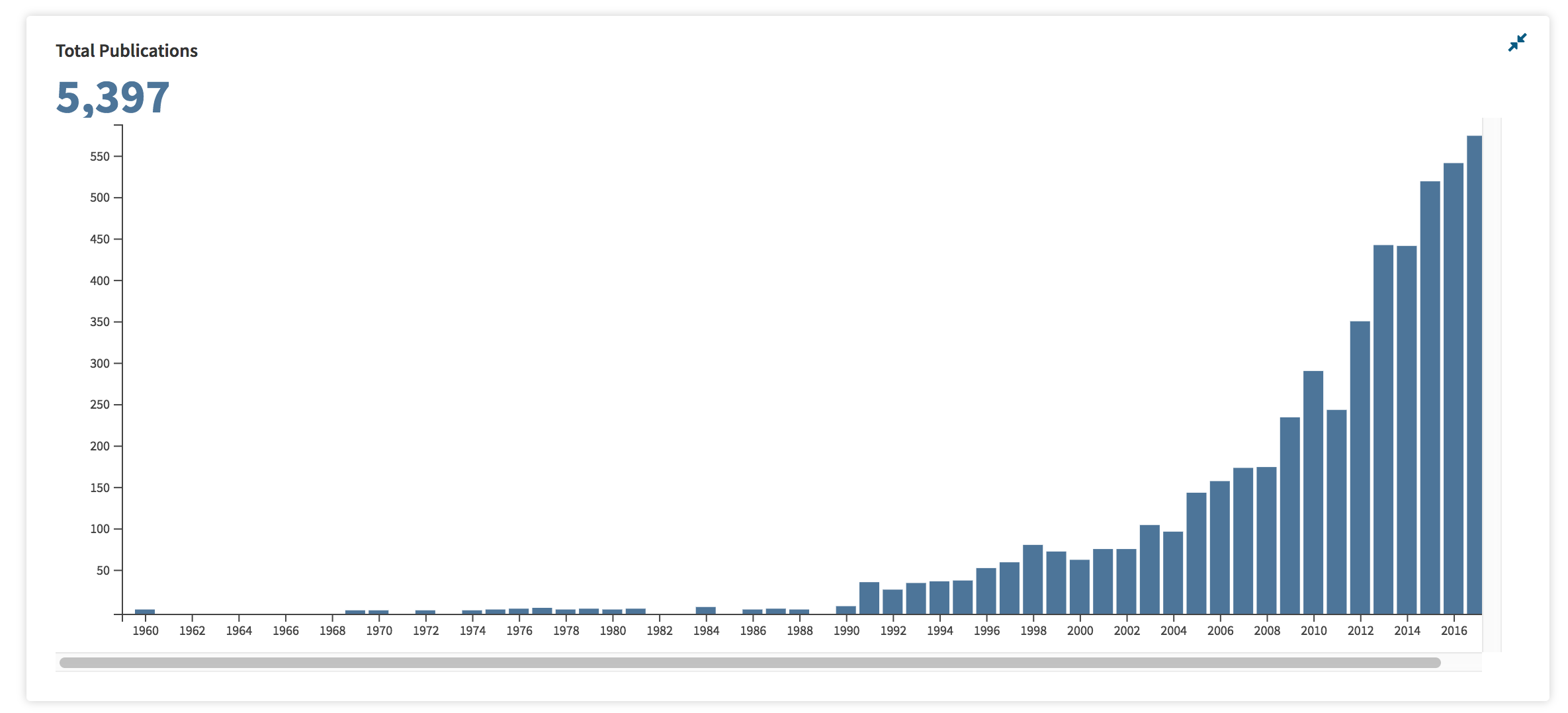


Figure 1. Papers published from 1960 – 2017 under the urban rainfall/ precipitation topical area per year as noted from Web of Science.

D. Meta-analysis results of subgroups

| 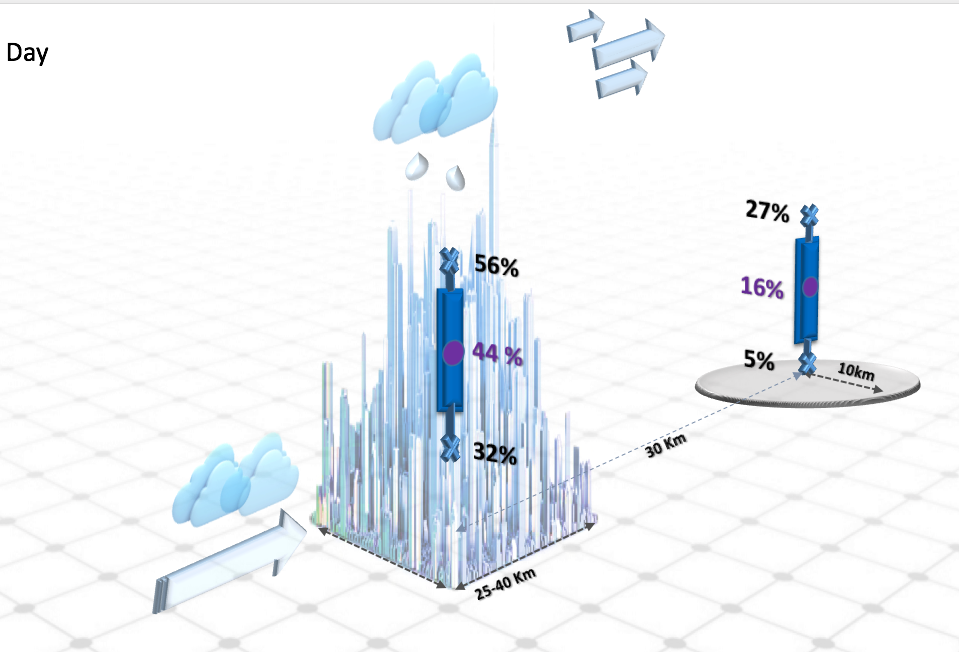 |
| --- |
| 1. Day |
| 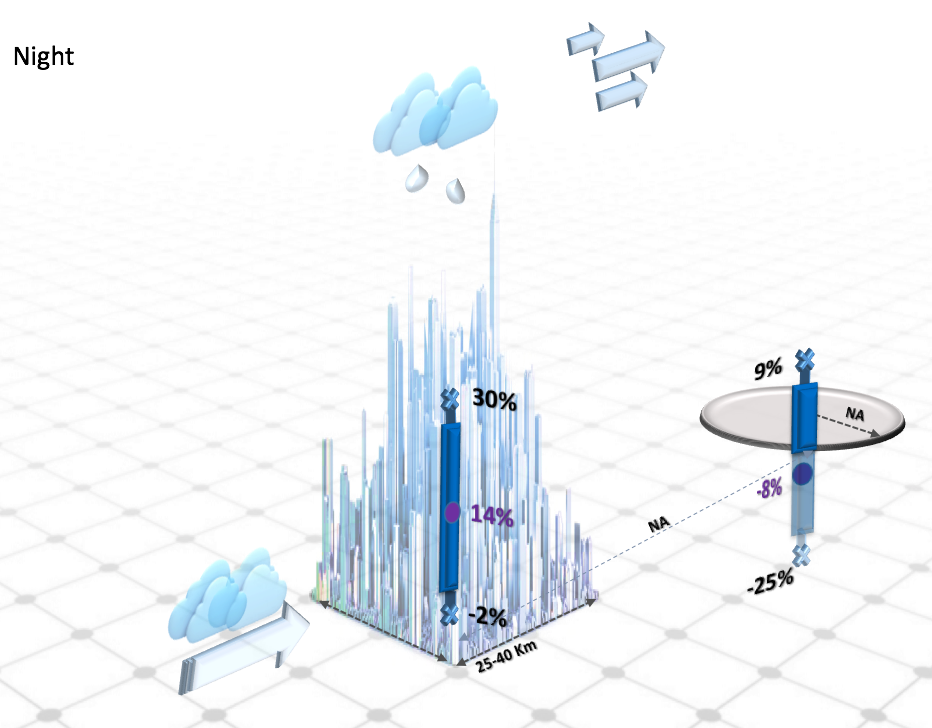 |
| 1. Night |

Figure 2. Meta-analysis results for day versus night (climatological studies only). During day time, a significant increase of precipitation over the city and downwind of the city is noted. During night time, there is an increase over the city while no precipitation change in the downwind. While the Urban Heat Island (UHI) information is not available, it can be assumed from the broader literature that nighttime conditions typically have a sustained UHI, and may be one of the reasons for the difference in the increased rainfall over versus downwind of the city.

| 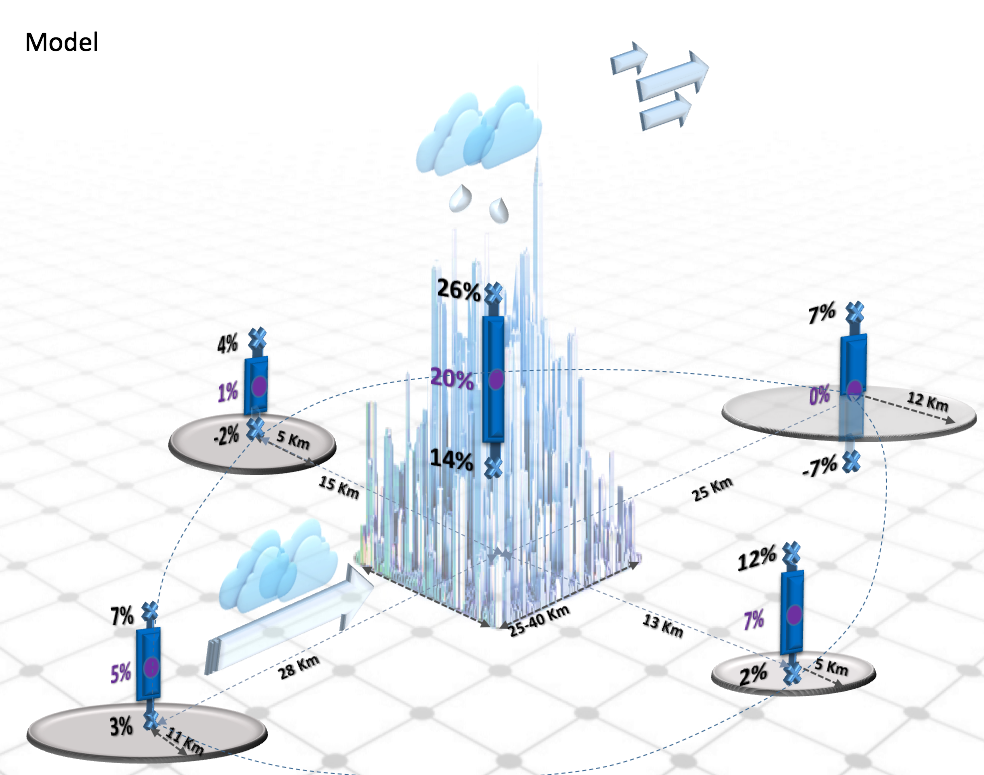 |
| --- |
| 1. Model |
| 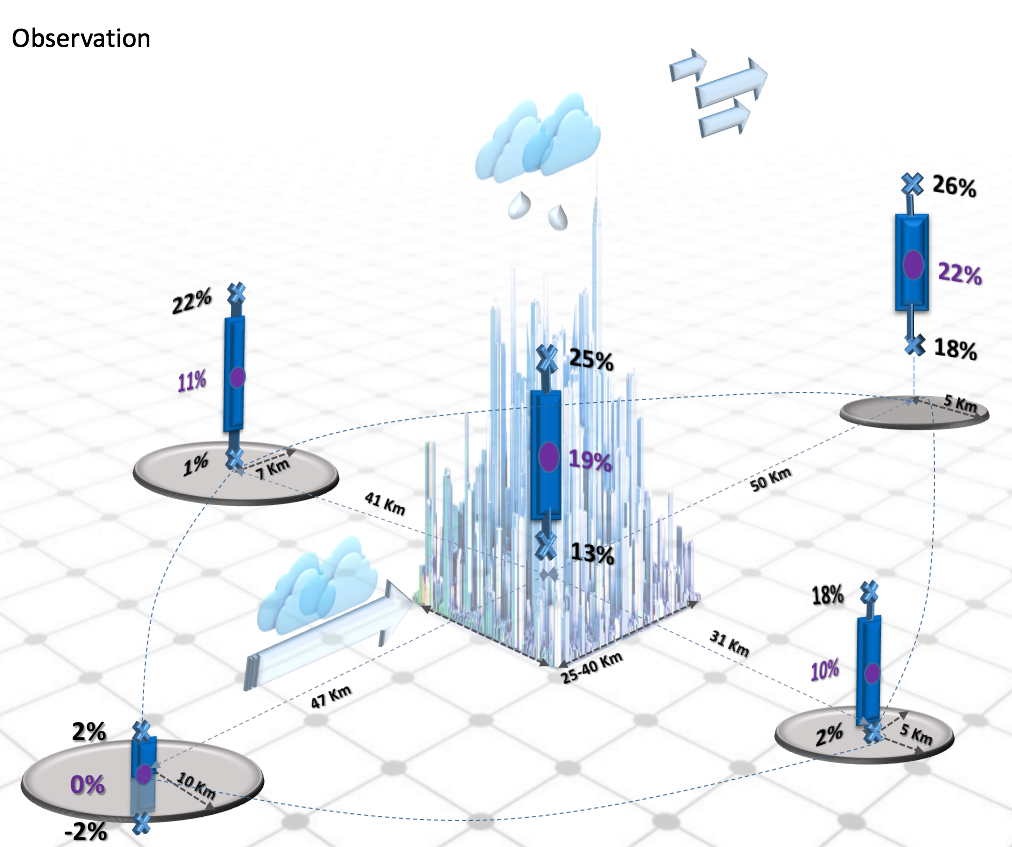 |
| 1. Observational |

Figure 3. Subgroup meta-analysis result for modeling versus observational studies (climatological studies only). Results show good agreement between the two, with both indicating precipitation increase in downwind and center of the city.

| 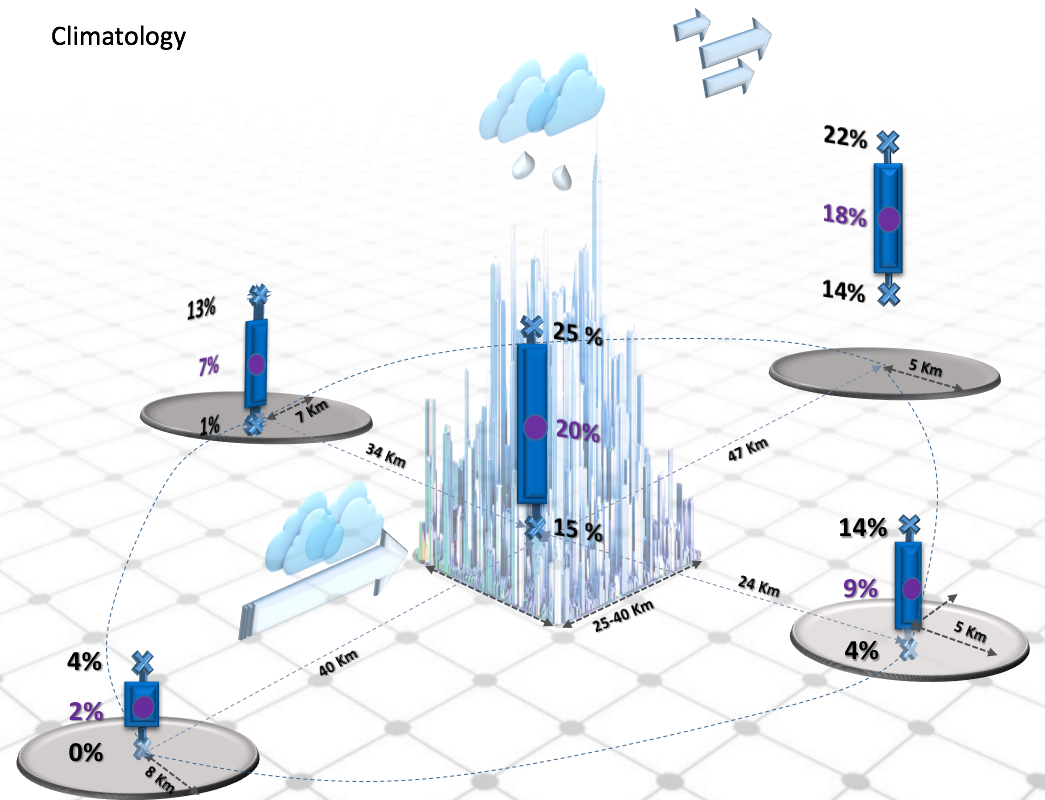 |
| --- |
| 1. Climatology |
| 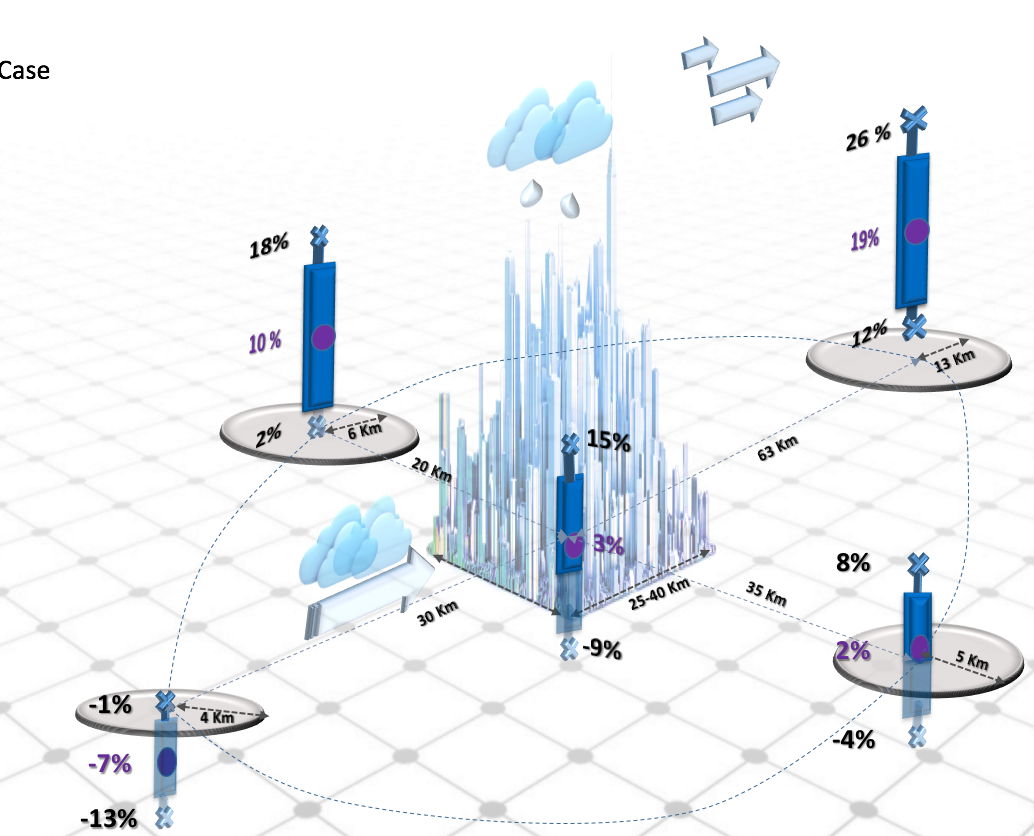 |
| 1. Case Studies |

Figure 4. Subgroup meta-analysis results: Climatology versus Case studies. The climatological studies show significant precipitation increases over the urban center as well as the downwind area, while in the case studies, a notable increase in the downwind and the left side of the storm.
